# Correlation between severe acute respiratory syndrome coronavirus 2 infection and serum anti-neutrophil cytoplasmic antibody formation

**DOI:** 10.3389/fimmu.2025.1593577

**Published:** 2025-05-16

**Authors:** Hao Huang, Yu-Jie Zang, Zheng-Guo Li, Qian Li, Chun-Yan Ma, Hai-Yuan Zhao, Hang Li

**Affiliations:** Department of Laboratory Medicine and Key Laboratory of Tumor Marker Detection, Kaifeng Central Hospital, Kaifeng, Henan, China

**Keywords:** anti-neutrophil cytoplasmic antibody, antinuclear antibody, indirect immunofluorescence assay, infection, severe acute respiratory syndrome coronavirus

## Abstract

**Objective:**

To investigate the impact of SARS-CoV-2 infection on the formation of antineutrophil cytoplasmic antibodies (ANCA).

**Methods:**

Serum samples from 154 patients infected with SARS-CoV-2 were collected and tested for ANCA and antinuclear antibodies (ANA) using indirect immunofluorescence assay (IFA). Enzyme-linked immunosorbent assay (ELISA) was used to confirm the target antigens in ANCA fluorescence-positive samples. Eighty-seven healthy individuals were selected as the control group. Statistical analysis was performed using SPSS 26.0 software, with P < 0.05 considered statistically significant.

**Results:**

The ANCA fluorescence positivity rate in the control group (1.1%) was not significantly different from that in COVID-19 positive patients (2.5%) (χ² = 0.574, P > 0.05). Among COVID-19 positive patients, 4 cases (2.5%) were ANCA fluorescence positive, while 10 cases (6.4%) were ANA positive. The difference between these two rates was not statistically significant (χ² = 2.694, P > 0.05).

**Conclusion:**

The Omicron variant of SARS-CoV-2 has minimal association with the formation of serum ANCA.

## 1 Introduction

Since December 2019, when pneumonia cases caused by the novel coronavirus (severe acute respiratory syndrome coronavirus 2 (SARS-CoV-2)) were first identified both domestically and internationally, SARS-CoV-2 has become a source of widespread concern. Research has demonstrated that the serum of individuals infected with COVID-19 contains various autoantibodies, including antinuclear antibodies (ANA), anti-Sjögren’s syndrome A (SSA) antibodies, anti-Ro-52 and anti-Ro-60 antibodies, antiphospholipid antibodies (APL), anti-interferon (IFN)-α and anti-IFN-1 antibodies, anti-interleukin (IL) antibodies, anti-chemokine antibodies, and anti-cardiolipin antibodies (1). Numerous studies have reported that these autoantibodies not only contribute to specific clinical manifestations in patients with COVID-19 but are also closely linked to disease severity (2–6).

Research has demonstrated that infections caused by certain viruses, bacteria, and fungi are associated with the formation of antineutrophil cytoplasmic antibodies (ANCA) (7). ANCA serves as a crucial laboratory marker for antineutrophil cytoplasmic antibody-associated vasculitis (AAV), with indirect immunofluorescence assay (IFA) being the primary detection method (8–10). Recent studies have identified multiple shared key genes between AAV and SARS-CoV-2 (11). However, there are currently no reports on ANCA formation in the serum of SARS-CoV-2-infected patients without AAV. This study employed IFA and enzyme-linked immunosorbent assay (ELISA) to detect ANCA and its spectrum in the serum of patients with SARS-CoV-2 infection to determine whether SARS-CoV-2 infection is associated with ANCA formation.

## 2 Participants and methods

### 2.1 Research participants

A random sampling method was employed to select 154 patients who tested positive for SARS-CoV-2 via oropharyngeal swab nucleic acid testing and visited Kaifeng Central Hospital between February and September 2024 as the study group (observation group). This group comprised 77 males and 77 females, with ages ranging from 17 to 98 years and a mean age of 70.7 years. The inclusion criteria were based on the Diagnosis and Treatment Protocol for Novel Coronavirus Pneumonia (Trial Version 8, Revised Edition) (12), which classified the patients into clinical categories as follows: 8 mild cases, 115 moderate cases, 14 severe cases, and 17 critical cases. Additionally, 87 healthy individuals who tested negative for SARS-CoV-2 were selected as the control group, including 26 males and 61 females, with ages ranging from 24 to 81 years and a mean age of 50.6 years. All study participants were screened to exclude those with antineutrophil cytoplasmic antibody-associated vasculitis. This study was approved by the Ethics Committee of Kaifeng Central Hospital (Approval No.: kt2024-013), and informed consent was obtained from all participants.

### 2.2 Methods

#### 2.2.1 ANCA detection

The Anti-Neutrophil Cytoplasmic Antibody IgG Detection Kit (Euroimmun, Germany) using an indirect IFA was employed to detect anti-neutrophil cytoplasmic antibodies. All procedures were performed manually in strict accordance with the kit’s instructions. The Eurostar III Plus fluorescence microscope (Euroimmun, Germany) was used for manual slide reading, with a titer of ≥ 1:10 considered positive. ANCA fluorescence results were classified as pANCA, cANCA, or atypical ANCA, following the relevant expert consensus (13).

#### 2.2.2 ANA detection

The Antinuclear Antibody IgG Detection Kit (Euroimmun, Germany) using an indirect IFA was employed to detect antinuclear antibodies. All procedures were performed manually in strict accordance with the kit’s instructions. The fluorescence pattern was interpreted based on the 2021 International Consensus on Antinuclear Antibody Fluorescence Pattern (ICAP) (14). The EUROSTAR III Plus fluorescence microscope (Euroimmun, Germany) was used for manual slide reading, with a titer of ≥ 1:100 considered positive.

#### 2.2.3 ANCA spectrum detection

The ANCA Profile IgG Detection Kit (Euroimmun, Germany) using an ELISA was employed to detect the ANCA spectrum, which included proteinase 3 (PR3), lactoferrin (LF), myeloperoxidase (MPO), elastase (EL), cathepsin G (CATG), and bactericidal/permeability-increasing protein (BPI). Manual procedures were strictly performed according to the kit’s instructions. Results were semi-quantitatively determined using the ratio calculation formula provided in the kit. For PR3 detection, a ratio × 1.4 < 1.0 was considered negative, 1.0 ≤ ratio × 1.4 < 2.0 was weakly positive, 2.0 ≤ ratio × 1.4 < 5.0 was positive, and ratio × 1.4 ≥ 5.0 was strongly positive. For the detection of other antigens, a ratio < 1.0 was negative, 1.0 ≤ ratio < 2.0 was weakly positive, 2.0 ≤ ratio < 5.0 was positive, and ratio ≥ 5.0 was strongly positive.

### 2.3 Statistical analysis

IBM SPSS Statistics 26 software was utilized for statistical analysis and description, with count data presented as case numbers or rates. Comparisons of rates were conducted using the χ² test or Fisher’s exact probability test, with a significance threshold of *p* < 0.05. Logistic regression analysis evaluates the predictive factors for ANCA formation.

## 3 Results

### 3.1 Comparison of ANCA positivity rates among COVID-19 patients of different severities

Using IFA to test 154 COVID-19 positive serum samples, 4 cases (2.5%) were ANCA positive. In the control group of 87 individuals, 1 case (1.1%) was ANCA positive. Comparison between these two groups yielded a χ² value of 0.574, with P > 0.05, indicating no statistically significant difference. Additionally, when comparing the ANCA positivity rates among COVID-19 patients of different severities, the χ² value was 0.990, with P > 0.05, indicating no statistically significant difference (see **Table 1**).

**Table 1:** Comparison of ANCA positivity rates among COVID-19 patients of different severities.

Disease classification	Positive casw(%)	Negative case(%)	χ2	P value
Mild	0(0)	8(5.2)	0.99	0.804
Moderate	4(2.5)	115(74.7)		
Severe	0(0)	14(9.1)		
Critical	0(0)	17(11.0)		
Total	4	150		
Control group	1(1.1)	86(98.9)	0.574	0.449

### 3.2 IFA detection of ANCA formation in 154 COVID-19 positive serums

**Figure 1:**
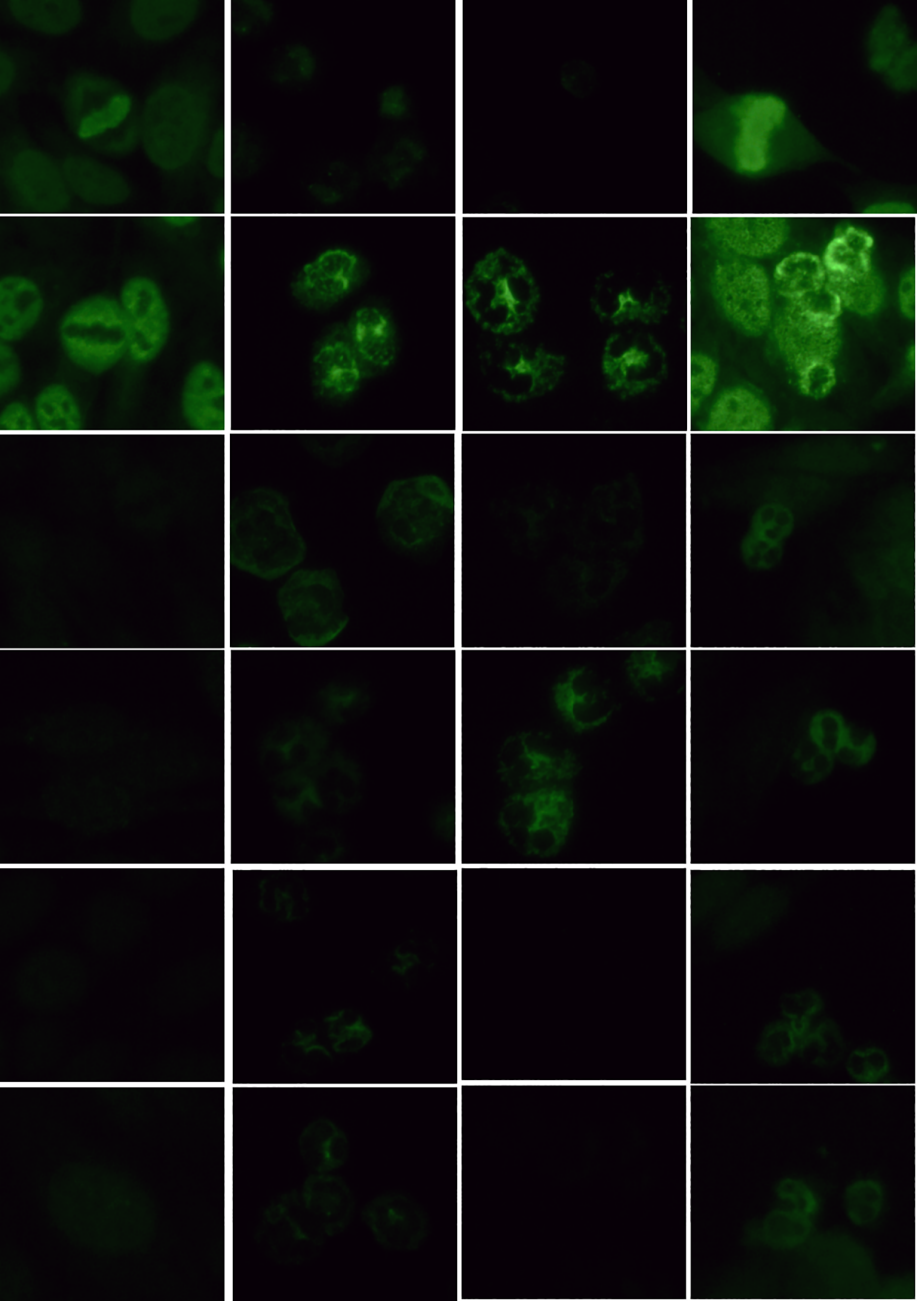
Detection of ANCA fluorescence patterns in COVID-19 nucleic acid-positive serum using IFA (40x objective lens). (1) HEp-2 cell substrate (dilution ratio 1:100); (2) granulocytes (ethanol-fixed) refers to ethanol-fixed human neutrophil granulocytes matrix (dilution ratio 1:10); (3) granulocytes (formaldehyde-fixed) refers to formaldehyde-fixed human neutrophil granulocytes matrix (dilution ratio 1:10); (4) HEp-2 + granulocytes refers to a mixed matrix of ethanol-fixed HEp-2 cells and human neutrophil granulocytes (dilution ratio 1:10).

Among the 4 cases (2.5%) that tested positive for ANCA fluorescence, the distribution was as follows: 1 case (0.6%) was pANCA with a titer of 1:100; 1 case (0.6%) was cANCA with a titer of 1:32; 1 case (0.6%) was atypical pANCA with a titer of 1:10; and 1 case (0.6%) was atypical ANCA with a titer of 1:10. Using IFA to test COVID-19 positive serum samples, 96.1% (148/154) were ANCA negative on ethanol-fixed substrate slides, while 1.9% (3/154) were pANCA-positive, 1.2% (2/154) were cANCA-positive, and 0.6% (1/154) were atypical ANCA-positive. On formaldehyde-fixed substrate slides, 3 cases (1.9%) tested positive. The fluorescence patterns are presented in **Figure 1**. All serum samples with fluorescence (5.2%, 6/115) belonged to patients with moderate cases. Comparing the ANCA fluorescence positivity rates among th**e** four groups, χ² = 1.345, P > 0.05, indicating no statistically significant difference.

Binary logistic regression analysis was performed to assess the predictive factors for ANCA formation in the observation group, considering variables such as age, gender, and comorbidities including diabetes mellitus, coronary artery disease, malignancy, cerebral infarction, renal insufficiency, and positive hepatitis B surface antigen. The P-values for all variables were > 0.05, indicating no significant impact (see **Table 2**).

**Table 2:** Logistic regression analysis of factors associated with ANCA formation.

Item	B	Standard Error	Wald	Significance	Exp(B)	95% Confidence Interval for EXP(B)	
						Lower	Upper
Age	0.007	0.038	0.034	0.854	1.007	0.935	1.085
Gender	1.212	1.213	0.998	0.318	3.359	0.312	36.208
Diabetes	-16.899	9852.771	0.000	0.999	0.000	0.000	.
Coronary Artery Disease	0.314	1.255	0.063	0.803	1.369	0.117	16.013
Malignancy	1.566	1.320	1.408	0.235	4.789	0.360	63.667
Cerebral Infarction	-17.341	8178.340	0.000	0.998	0.000	0.000	.
Renal Insufficiency	-17.695	14882.113	0.000	0.999	0.000	0.000	.
Hepatitis B Surface Antigen Positive	-15.617	15827.706	0.000	0.999	0.000	0.000	.
Constant	59.546	25218.170	0.000	0.998	72554009491559390000000000.000		

### 3.3 Detection of ANA in 154 COVID-19 positive serum samples using IFA

**Figure 2:**
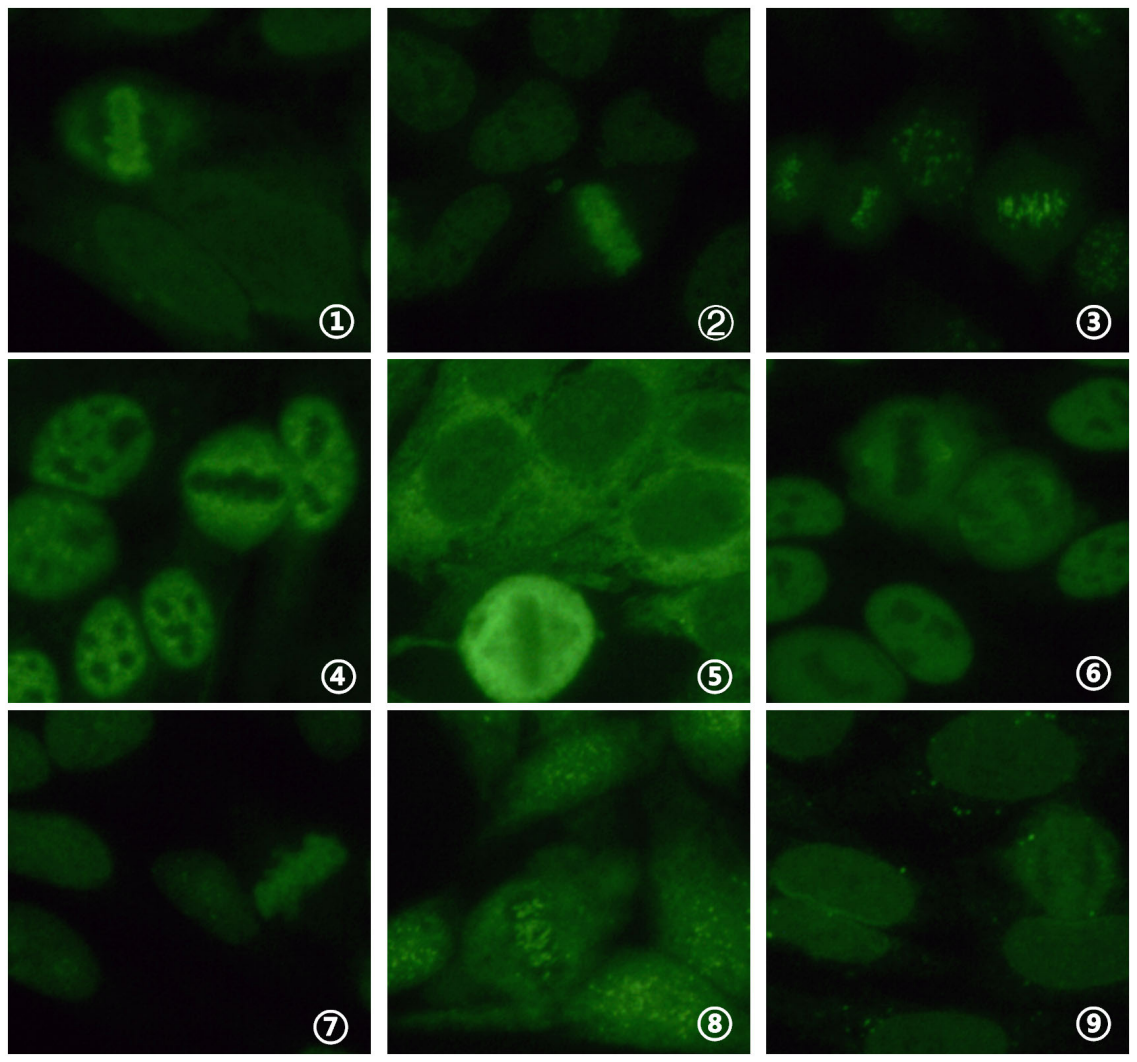
Detection of ANA fluorescence patterns in COVID-19 nucleic acid-positive serum using IFA (40x objective lens). (1) homogeneous nuclear pattern (AC-1); (2) dense fine speckled nuclear pattern (AC-2); (3) centromere pattern (AC-3); (4) coarse speckled nuclear pattern (AC-5); (5) dense fine speckled cytoplasmic pattern (AC-19); (6) NuMA-type spindle apparatus pattern (AC-26); (7) homogeneous nuclear pattern (AC-1) + fine speckled nuclear pattern (AC-4); (8) centromere pattern (AC-3) + fine speckled nuclear pattern (AC-4); (9) homogeneous nuclear pattern (AC-1) + punctate nuclear membrane pattern (AC-12).

Ten cases (6.4%) tested positive for antinuclear antibody fluorescence, with titers ranging from 1:100 to 1:1000 among 154 COVID-19-positive serum samples. Among these, single nuclear patterns included 0.1% (1/10) with a homogeneous nuclear pattern (AC-1), 0.1% (1/10) with a dense fine speckled nuclear pattern (AC-2), 0.2% (2/10) with a centromere pattern (AC-3), 0.1% (1/10) with a coarse speckled nuclear pattern (AC-5), 0.1% (1/10) with a dense fine speckled cytoplasmic pattern (AC-19), and 0.1% (1/10) with a NuMA-type spindle apparatus pattern (AC-26). Composite nuclear patterns included 0.1% (1/10) with a homogeneous nuclear pattern (AC-1) + fine speckled nuclear pattern (AC-4), 0.1% (1/10) with a centromere pattern (AC-3) + fine speckled nuclear pattern (AC-4), and 0.1% (1/10) with a homogeneous nuclear pattern (AC-1) + punctate nuclear membrane pattern (AC-12). The fluorescence patterns are presented in **Figure 2**. A comparison of the 10 cases (6.4%) testing positive for antinuclear antibody with the 4 cases (2.5%) testing positive for ANCA yielded a p value of 0.101 (> 0.05), χ² = 2.694, indicating no statistically significant difference. Among moderate cases, 7.8% (9/115) were antinuclear antibody positive, while among severe cases, 7.1% (1/14) were antinuclear antibody positive.

### 3.4 Confirmation of ANCA spectrum in samples positive on ethanol or formaldehyde-fixed substrate slides

**Figure 3:**
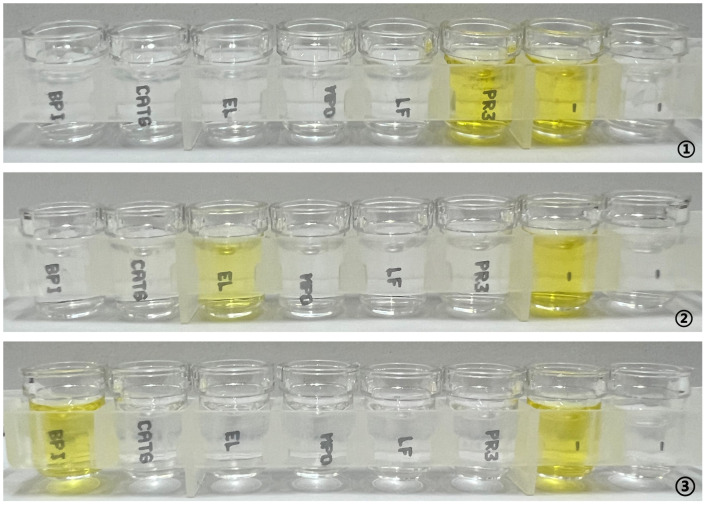
ANCA spectrum detection in samples positive for ethanol- or formaldehyde-fixed substrate slides using ELISA. (1) anti-PR3 antibody ratio strongly positive; (2) anti-elastase antibody ratio positive; (3) anti-bactericidal/permeability-increasing protein antibody ratio positive.

An analysis of 154 COVID-19-positive serum samples identified six cases exhibiting fluorescence on ethanol-fixed or formaldehyde-fixed substrate slides. ANCA spectrum testing revealed that 16.7% (1/6) tested strongly positive for anti-PR3 antibody, 16.7% (1/6) were positive for anti-elastase antibody, and 33.3% (2/6) were weakly positive and positive for anti-BPI antibody, respectively. Anti-lactoferrin antibody, anti-MPO antibody, and anti-cathepsin G antibody were not detected. **Figure 3** presents the positive ANCA spectrum results.

### 3.5 Confirmation of ANCA spectrum in severe and critical cases

Serum samples from 14 severe and 17 critical patients were tested for the ANCA spectrum. In the severe cases, 14.3% (2/14) tested positive for anti-bactericidal/permeability-increasing protein (BPI) antibodies, while detection rates for other antibodies remained at 0. In the critical cases, no antibodies were detected. Due to the limited number of samples in both groups, only the positivity rates were calculated without statistical analysis. The distribution of ANCA fluorescence and ANCA spectrum positivity is shown in **Table 3**.

**Table 3:** Distribution of ANCA spectrum positivity.

ANA fluorescence pattern	Granulocytes (Ethanol)	Granulocytes (Formaldehyde)	ANCA interpretation result	ANCA spectrum
AC-1	pANCA	negative	negative	BPI
AC-5	pANCA	cANCA	pANCA	Elastase
negative	aytpicalANCA	cANCA	aytpicalANCA	negative
negative	cANCA	cANCA	cANCA	PR3
negative	pANCA	negative	aytpicalANCA	negative
negative	cANCA	negative	negative	BPI
negative	negative	negative	negative	BPI
AC-3+AC-4	negative	negative	negative	BPI

Among the 4 samples with positive ANCA fluorescence interpretations, 2 had positive ANCA spectrum results. In the 2 samples that only showed fluorescence on ethanol-fixed substrate slides and were interpreted as ANCA negative, BPI was detected in both. Two samples had only BPI positivity with ANCA negativity.

## 4 Discussion

In the formation of ANCA, viruses such as human immunodeficiency virus, hepatitis B virus, hepatitis C virus, parvovirus B19, Epstein-Barr virus, arbovirus, and Ross River virus have all been proven to induce ANCA positivity, yet their clinical diagnostic significance is limited (7). Previous studies in China have found that the positivity rate of ANCA in the general population is 2.29% (15), which is comparable to the ANCA positivity rate of 2.5% observed in COVID-19 patients in our study. It is believed that the formation of ANCA during infection may involve several potential mechanisms: autoimmune reactions triggered by the complementarity of microbial peptides and self-antigens; upregulation of self-antigen genes due to epigenetic silencing and antigen complementarity; molecular mimicry between bacterial antigens and self-antigens; the formation of neutrophil extracellular traps (NETs) and the ensuing immune processes, including the production of ANCA; and the interaction between bacterial components and Toll-like receptors (TLRs), leading to the formation of immune reaction mediators that may trigger the production of ANCA (16).

Gregg E et al. (17) found that the positivity rate of ANA in the general US population is 11%–15.9%, while in China, the positivity rate of ANA in the general population ranges from 7.4% to 13.5% (18). In our study, the positivity rate of ANA in COVID-19 patients (6.4%) is not significantly different from that in the general Chinese population.One study analyzed 12 types of autoantibodies in 21 patients with severe and critical COVID-19 infections, reporting detection rates of 50% for ANA, 25% for anti-Ro-60 antibodies, 20% for anti-Ro-52 antibodies, 5% for anti-DNA topoisomerase I (Scl-70) antibodies, and 5% for anti-U1 ribonucleoprotein (U1-RNP) antibodies (4). Findings from this study indicate whether ANCA formation was detected using IFA in serum samples from patients having mild, moderate, severe, and critical COVID-19. Alternatively, ELISA was used in fluorescence-patterned serum samples and severe and critical cases, and detection rates were considerably lower than those reported in prior research on other autoantibodies (4). Pascolini et al. identified autoantibodies in 33 COVID-19 patients and found 33% were ANA positive (5). In contrast, the overall ANA positivity rate in COVID-19 serum samples in this study was 6.4%, with a positivity rate of only 3.2% in severe and critical cases, differing from previous ANA detection rates in patients with COVID-19 (4, 5).

Possible reasons for these findings include differences in study periods and viral strains. The earlier studies were conducted during the first half of 2020 at the onset of the SARS-CoV-2 pandemic and examined the early wild-type strain. Research from 2023 demonstrated that changes in innate immune phenotypes and epigenetic programming of hematopoietic stem cells and progenitor cells persisted for months to a year after SARS-CoV-2 infection, influencing post-COVID immunity and recovery (19). In contrast, this study focused on the Omicron variant JN.1 from the first half of 2024 in China (20). By this time, four years had passed since the initial outbreak, most individuals had COVID antibodies, and no literature has reported the effects of COVID-19 infection on the body after 2023. The production of antibodies, recovery processes, and the virulence of variant strains may have significantly declined compared to the wild-type strain, which could contribute to the lower autoantibody production.

Additionally, studies on human leukocyte antigen (HLA) alleles in patients with COVID-19 have established links between HLA variations and disease severity (21, 22). Research indicates that different HLA types exhibit varying capacities to bind to the virus. An analysis of HLA allele frequency distribution in 99 Italian patients identified significant associations between SARS-CoV-2 susceptibility and HLA-DRB1*15:01 and HLA-DQB1*06:02 (23). Globally, only 0.63% of the population carries the HLA-B*15:03 allele, which confers relative resistance to SARS-CoV-2 infection. The prevalence of this genotype’s presence in China is at a medium level on a worldwide ranking (24). Therefore, genetic factors related to HLA allele carriage may also influence autoantibody formation.

Simultaneous detection of ANCA using both IFA and ELISA can enhance the specificity of disease diagnosis and broaden its clinical application. ANCA serves as a specific serological marker for ANCA-associated vasculitis, with target antigens MPO and PR3 playing a major role in the diagnosis of microscopic polyangiitis and granulomatosis with polyangiitis, respectively. Other target antigens are rarely or never detected in autoimmune diseases (25). Our study found that among the two specimens positive by both IFA and ELISA, one case was pANCA and elastase positive, clinically diagnosed with gallbladder malignancy with liver and lymph node metastases; the other case was cANCA and PR3 positive, diagnosed with infectious fever. The application of ANCA is not limited to vasculitis but can occur due to environmental exposure, medication use, or various disease processes. Conditions associated with ANCA beyond vasculitis include chronic inflammatory diseases, other autoimmune disorders, malignancies, leukemia, and infections (7). Since both ANCA-positive cases in our study were linked to conditions unrelated to vasculitis, the findings do not indicate a direct association between ANCA formation and SARS-CoV-2 infection. Whether these two patients will later develop AAV or other autoimmune diseases requires long-term follow-up.

For the two samples in our study that were interpreted as atypical ANCA by fluorescence but did not show detectable target antigens, research has highlighted inconsistencies between ANCA detection using IFA and target antigen confirmation. These discrepancies may arise from various factors, including the choice of detection method, differences in sample processing, and changes in antibody specificity. Additionally, the presence of ANA may interfere with ANCA detection, leading to false-positive results (26). Our study employed a multi-matrix combination for ANCA detection, effectively excluding ANA interference. However, the possibility of unknown target antigens cannot be ruled out.

Studies have shown that patients with negative IFA results but positive ELISA findings are often linked to non-vascular inflammatory tissue diseases, hypertension, and heart disease (27–29). This observation suggests that these patients may have complex immune responses and inflammatory mechanisms. Chronic inflammation is common in individuals with hypertension and heart disease, potentially leading to abnormal immune system responses that influence IFA and ELISA results. BPI, a soluble protein in neutrophil granules, plays a role in defense against gram-negative bacteria. Anti-BPI antibodies are frequently detected in patients with cystic fibrosis and Pseudomonas infections (30).

In our study, only two severe cases matched the above reports, and no direct evidence confirms a link between BPI formation and COVID infection. In summary, the impact of SARS-CoV-2 on the general population has gradually weakened since its outbreak. However, whether long-term immune system abnormalities persist requires further investigation.

The limitations of this study mainly lie in the limited sample size and the single source of samples. This leads to insufficient statistical power, which may affect the accurate assessment of the association between SARS-CoV-2 infection and the formation of serum ANCA, and makes it difficult to comprehensively reflect the overall situation of patients during the prevalence of different variants in different regions, thus limiting the generalizability of the study results. Future studies should increase the sample size and cover patients from more regions and infected with different variants to enhance statistical power and verify the findings of this study.

## 5 Conclusion

Only two cases in severe and critical patients matched the above reports, and no direct evidence confirms a link between BPI formation and COVID infection. In summary, the impact of SARS-CoV-2 on the general population has gradually weakened since its outbreak. However, whether long-term immune system abnormalities persist requires further investigation.

## Data Availability

The original contributions presented in the study are included in the article/supplementary material. Further inquiries can be directed to the corresponding author.
